# Ageism in Training: Measuring Attitudes Among Medical, Social Work, and PA Students

**DOI:** 10.1093/geroni/igaf122.1921

**Published:** 2025-12-31

**Authors:** Euna Cho, Diane Martin

**Affiliations:** University of Maryland School of Medicine, Baltimore, Maryland, United States; University of Maryland, Baltimore County, Baltimore, Maryland, United States

## Abstract

Ageism in healthcare negatively affects older adults’ health outcomes and increases healthcare costs. Research shows that healthcare professionals such as physicians and social workers hold ageist attitudes. However, there is limited research on the attitudes toward older adults of healthcare students. This study aimed to assess ageist attitudes among first- and second-year graduate and professional students. Fifty-four students (14 medical, 14 social work, and 26 physician assistant) completed the Fraboni Scale of Ageism (FSA) — a validated tool measuring three components of ageist attitudes: Antilocution (negative language; 10 items); Discrimination (unequal treatment; 9 items); Avoidance (social distancing; 10 items). Ageist attitudes were present across healthcare student disciplines, with no significant differences between groups. While the three groups scored in the low-range for antilocution, they all notably scored in the mid-range for discrimination (medical students M = 15.33, SD = 2.09; physician assistant students M = 15.52, SD = 2.94, social work students M = 16.1, SD = 3.21), suggesting a moderate belief in limiting older adults’ roles and abilities. Additionally, avoidance scores for medical students (M = 17.20; SD = 4.41) and physician assistant students (M = 17.45; SD = 3.85) indicate a moderate tendency to distance themselves from older adults. These findings illustrate the need to design and incorporate healthcare education and training that challenges age-related stereotypes, reduces social distancing behaviors, and promotes a more accurate and compassionate understanding of aging and older adults across professional healthcare disciplines.

